# State-of-the-Art and Future Directions in Organ Regeneration with Mesenchymal Stem Cells and Derived Products during Dynamic Liver Preservation

**DOI:** 10.3390/medicina58121826

**Published:** 2022-12-12

**Authors:** Nicola De Stefano, Alberto Calleri, Victor Navarro-Tableros, Federica Rigo, Damiano Patrono, Renato Romagnoli

**Affiliations:** 1 General Surgery 2U—Liver Transplant Unit, Azienda Ospedaliero Universitaria Città della Salute e della Scienza di Torino, University of Torino, 10126 Turin, Italy; 2 Gastrohepatology Unit, Azienda Ospedaliero Universitaria Città della Salute e della Scienza di Torino, University of Torino, 10126 Turin, Italy; 3 2i3T, Società per la Gestione dell’incubatore di Imprese e per il Trasferimento Tecnologico, University of Torino, 10126 Turin, Italy

**Keywords:** liver machine perfusion, liver regeneration, mesenchymal stem cells, extracellular vesicles, liver transplantation

## Abstract

Transplantation is currently the treatment of choice for end-stage liver diseases but is burdened by the shortage of donor organs. Livers from so-called extended-criteria donors represent a valid option to overcome organ shortage, but they are at risk for severe post-operative complications, especially when preserved with conventional static cold storage. Machine perfusion technology reduces ischemia-reperfusion injury and allows viability assessment of these organs, limiting their discard rate and improving short- and long-term outcomes after transplantation. Moreover, by keeping the graft metabolically active, the normothermic preservation technique guarantees a unique platform to administer regenerative therapies ex vivo. With their anti-inflammatory and immunomodulatory properties, mesenchymal stem cells are among the most promising sources of therapies for acute and chronic liver failure, but their routine clinical application is limited by several biosafety concerns. It is emerging that dynamic preservation and stem cell therapy may supplement each other if combined, as machine perfusion can be used to deliver stem cells to highly injured grafts, avoiding potential systemic side effects. The aim of this narrative review is to provide a comprehensive overview on liver preservation techniques and mesenchymal stem cell-based therapies, focusing on their application in liver graft reconditioning.

## 1. Introduction

Machine perfusion (MP) of the liver can reduce ischemia-reperfusion injury (IRI) and improve outcomes of liver transplantation (LT), especially when grafts from extended criteria donors (ECDs) are used [1]. The benefits of MP have been demonstrated in several retrospective studies [2,3,4,5] and randomized clinical trials [6,7,8,9,10,11]. Many trials are currently ongoing and will likely confirm MP as a standard-of-care in LT, especially for ECD procedures [12].

Despite the great success of MP in improving preservation and utilization of liver grafts, a consistent percentage of organs are still considered too damaged to be transplanted. Therefore, innovative approaches aimed at repairing severely injured organs may further increase the number of available grafts and allow LT for patients who might benefit from LT but are currently excluded due to organ shortage. 

Thanks to their anti-inflammatory and immunomodulatory effects, mesenchymal stem cells (MSCs) may represent one of these approaches. MSCs are among the most widely studied cell types [13] and have been tested in a variety of diseases, ranging from acute liver failure to myocardial and cerebral infarction [14,15]. Based on their multipotency, MSCs are able to migrate towards injured sites and promote tissue repair by either long-term engraftment and differentiation or, most relevantly, paracrine mechanisms [14,15]. Indeed, MSCs secrete a large variety of growth factors, cytokines and extracellular vesicles (EV) that are important mediators of cell-to-cell communication and can modulate the phenotype and behavior of their targets, being the most responsible actors of the biological responses related to MSC administration [15]. In LT, MSCs have raised attention due to the possibility of reducing IRI and inducing transplant tolerance, but their routine application is hampered by short cell survival, the considerable high doses required to obtain a tangible biological effect, unspecific biodistribution and the risk of adverse events [16]. 

**Figure 1:**
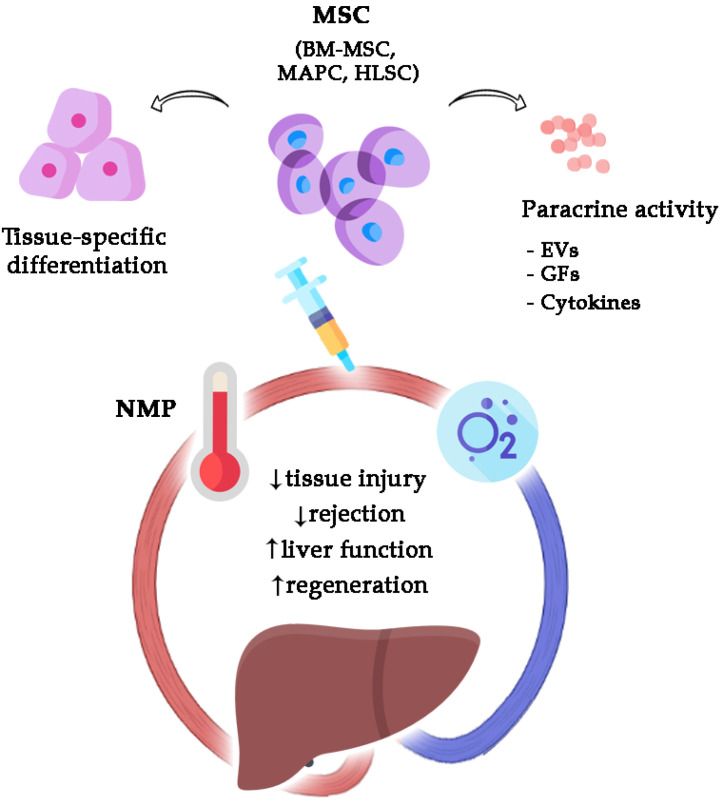
Biological activity of MSC-based therapies administered during liver NMP.

With the current progress of dynamic organ preservation, it is emerging that MP could serve as a platform to administer MSCs to damaged organs while avoiding the pitfalls of systemic administration (Figure 1). Despite the fact that this combined approach is still in a preclinical stage, important milestones have already been reached in liver, lung and kidney reconditioning, leading to the translation from rodent to large-size models [17]. 

The aim of this narrative review is providing an overview on current evidence on MSC-based therapies for hepatic diseases, focusing on their application during liver graft preservation. 

## 2. Materials and Methods

The Medline (PubMed) database was accessed on 30 June 2022 and searched for (‘liver AND machine perfusion’) OR (‘mesenchymal stem cell’ AND ‘liver injury’), with no time restrictions, retrieving 1727 articles. The literature review was performed by 4 authors (NDS, AC, FR and VNT) and any disagreement was resolved by consensus. Titles were screened to select potential relevant studies, initially including 580 articles. Next, abstracts of the selected items were screened according to the inclusion and exclusion criteria, leading to 66 articles being eligible for full-text review. A total of 33 additional articles were identified by manual cross-checking among the cited references and 6 recently published papers were added during the revision process, resulting in 105 included articles. Inclusion criteria were clinical and preclinical peer reviewed studies reporting on the use of machine perfusion technology in the liver transplantation setting and/or the use of mesenchymal stem cell-based therapies in liver disease, with no species, age or sex restriction. Case reports, letters to the editor and publications with no full-text available or published in languages other than English were excluded.

## 3. Results

### 3.1. Machine Perfusion of the Liver as a Dynamic Platform for Graft Reconditioning

MP devices were developed in the past century during the pioneer era of solid organ transplantation [18,19], but were soon replaced by static cold storage (SCS) that at that time, also due to the limits of available technology, resulted as cheaper, safer and easier to use. Currently, however, the suboptimal results obtained with ECD grafts preserved by SCS have generated a renewed interest in MP. Indeed, compared to SCS, MP can substantially improve outcomes of LT performed using grafts from ECDs by reducing IRI, allowing real-time viability testing and serving as a platform for organ reconditioning prior to transplantation [20,21,22]. 

Supported by a solid body of preclinical research, two landmark studies of the last decade successfully reported the first applications of MP technology in clinical LT [23,24]. Although indications and modalities remain heterogeneous [25], MP has progressively gained widespread adoption and it is now perceived as an essential tool in LT with grafts from ECDs (Table 1).

Depending on perfusion temperature, MP can be classified as normothermic machine perfusion (NMP, 37 °C), sub-normothermic machine perfusion (20–25 °C) or hypothermic machine perfusion (HMP, 10–12 °C). Graft perfusion can start ‘in situ’ during organ retrieval, as in the case of normothermic regional perfusion, or ‘ex situ’ after organ procurement; in the latter case, MP can be started at the donor hospital using portable devices (upfront MP or MP preservation) or at the recipient hospital after a period of SCS (end-ischemic MP) [26]. Combined sequential approaches are also possible for complex cases, such as NRP followed by end-ischemic MP [27,28].

The main goal of MP is the reduction of IRI. Briefly, the IRI cascade is initiated by ATP depletion and accumulation of NADH and succinate during cold ischemia, resulting in the abrupt release of mitochondrial-derived reactive oxygen species (ROS) upon graft reperfusion, which ultimately activates several pathways leading to cell death [29]. This process is even more enhanced in ECD livers, which poorly tolerate ischemia because of their impaired metabolic status [30]. Dynamic preservation can reduce or even prevent IRI by continuously providing oxygen and nutrients. In particular, hypothermic oxygenated MP restores the electron transport chain and ATP synthesis, promoting mitochondrial reconditioning and limiting ROS production upon reperfusion [29,31,32]. In contrast to HMP, NMP aims at reproducing a physiological environment for liver preservation. During NMP, cellular metabolism is maintained, allowing restoration of ATP production and mitochondrial recovery [29]. In addition, NMP modulates liver immune response towards an anti-inflammatory and pro-regenerative pattern [33].

The possibility to test graft quality and function in real-time is another important advantage of MP over SCS. Viability assessment makes the whole allocation process more informed, objective and safe, allowing utilization of grafts that would otherwise be discarded. Although viability assessment has long been considered possible only during NMP [20,21,34], assessment of liver function and injury is possible also during HMP [35,36,37,38,39].

**Table 1:** Clinical studies involving MP technology in LT.

Author	Study Type	MP	Donor	Endpoints	Outcomes
Guarrera et al., 2010 [23]	Clinical	HMP vs. SCS	DBD	▪ PNF, EAD, graft/patient survival at 1 mo and 1 y ▪ Biliary and vascular complications, serum surrogates, hospital-length of stay	↓ EAD ↓ hospital-length of stay, ALT, creatinine, bilirubin
Ravikumar et al., 2016 [24]	Clinical	NMP vs. SCS	DBD, DCD	▪ Graft survival at 1 mo ▪ Graft/patient survival, serum surrogates, EAD, ICU- and hospital-length of stay	↓ Peak AST
Van Rijn et al., 2017 [5]	Clinical	HOPE vs. SCS	DCD	▪ Graft survival at 6 mo ▪ Graft/patient survival at 1 y, EAD, ICU- and hospital-length of stay, biliary complications, serum surrogates	↓ ALT, GGT, ALP, bilirubin
Nasralla et al., 2018 [9]	Clinical (RCT)	NMP vs. SCS	DBD, DCD	▪ Peak AST ▪ Viability assessment, PRS, PNF, EAD, ICU- and hospital-length of stay, RRT, cholangiopathy on MRCP at 6 mo, graft/patient survival at 1 y	↓ Peak AST ↓ Discard rate, PRS, EAD
Ghinolfi et al., 2019 [11]	Clinical (RCT)	NMP vs. SCS	DBD	▪ Graft/patient survival at 6 mo ▪ Liver biopsies, peak ALT, biliary complications	↓ IRI-related features on tissue samples
Schlegel et al., 2019 [4]	Clinical	HOPE-DCD vs. SCS-DCD vs. SCS-DBD	DBD, DCD	▪ End-LT lactate, day 1 INR, peak ALT, ICU- and hospital-length of stay, biliary and vascular complications, PNF, CCI, Clavien Dindo complications, graft/patient survival (focus on non-tumor-related graft loss)	HOPE-DCD = SCS-DBD HOPE-DCD > SCS-DCD (↓ End-LT lactate, day 1 INR, hospital-length of stay, biliary complications, PNF, graft survival)
Patrono et al., 2019 [2]	Clinical	HOPE vs. SCS	DBD	▪ Peak AST and ALT, end-LT lactate, ICU- and hospital-length of stay, EAD, AKI, PRS, acute rejection rate, Clavien Dindo complications, biliary complications at 6 mo, graft/patient survival	↓ PRS, AKI, EAD, peak AST and ALT
Jassem et al., 2019 [33]	Clinical	NMP vs. SCS	DBD	▪ Gene profiling, biochemistry and histology	↓ Peak AST, IFN-γ- and IL-17-producing T cells, necrosis, apoptosis, neutrophil infiltration
Muller et al., 2019 [35]	Clinical	HOPE	ECD, DCD	▪ Viability assessment [within 30 min of hypothermic machine perfusion <10,000 units FMN in perfusate]	FMN predicts early graft loss, EAD, L-GrAFT, hospital-length of stay, kidney function, overall complications
Mergental et al., 2020 [21]	Clinical	NMP	Declined ECD	▪ Viability assessment [perfusate lactate < 2.5 mmol/L or evidence of bile production + at least 2 of the following: 1) pH > 7.3; 2) stable vascular flows (hepatic artery flow >150 mL/min and portal vein flow >500 mL/min); 3) homogeneous perfusion and soft consistency	Utilization rate: 71% PNF: 0% Ischemic cholangiopathy: 18%
Patrono et al., 2020 [36]	Clinical	HOPE	ECD	▪ Viability assessment [perfusate parameters: AST, ALT, LDH, glucose, pH]	Perfusate parameters correlate with macrovesicular steatosis and EAD, not on graft survival
Czigany et al., 2021 [7]	Clinical (RCT)	HOPE vs. SCS	DBD, ECD	▪ Peak ALT ▪ Clavien Dindo complications, CCI, EAD, ICU- and hospital-length of stay	↓ Peak ALT ↓ CCI, ICU- and hospital-length of stay
Van Rijn et al., 2021 [6]	Clinical (RCT)	HOPE vs. SCS	DCD	▪ Symptomatic NAS incidence at 6 mo ▪ PRS, PNF, EAD, hospital-length of stay	↓ NAS at 6 months ↓ PRS, EAD
Rayar et al., 2021 [38]	Clinical	HOPE vs. SCS	ECD	▪ EAD, PNF ▪ Blood units, PRS, serum surrogates, AKI, biliary complications at 1 y, ICU- and hospital-length of stay, Clavien Dindo complications, graft/patient survival at 1 y, economic impact	↓ AST, ALT, lactate, creatinine, ICU- and hospital-length of stay Same cost
Markmann et al., 2022 [10]	Clinical (RCT)	NMP vs. SCS	DBD, ECD, DCD	▪ EAD ▪ Viability assessment, PRS, biliary complications, overall patient survival	↓ EAD ↓ Discard rate, biliary complications, IRI-related features on tissue samples
Ravaioli et al., 2022 [8]	Clinical (RCT)	HOPE vs. SCS	ECD	▪ EAD ▪ Graft/patient survival, EASE score, hospital-length of stay, PNF, PRS, biliary and vascular complications	↓ EAD ↓ re-LT
Hann et al., 2022 [34]	Clinical	NMP	Declined ECD	▪ Viability assessment [perfusate lactate < 2.5 mmol/L or evidence of bile production + at least 2 of the following: 1) pH > 7.3; 2) stable flows (hepatic artery flow >150 mL/min and portal vein flow >500 mL/min); 3) homogeneous perfusion and soft consistency	Utilization rate: 72% PNF: 0% Ischemic cholangiopathy: 7%
Patrono et al., 2022 [3]	Clinical	HOPE vs. SCS	DBD, ECD	▪ EAF ▪ End-LT lactate, L-GrAFT, ICU- and hospital-length of stay, AKI, CCI, Clavien Dindo complications, biliary complications, graft/patient survival	↓ EAF ↓ CCI, Clavien Dindo complications, graft/patient survival
Patrono et al., 2022 [37]	Clinical	HOPE	ECD, DCD	▪ Viability assessment [microdialysate and perfusate levels of glucose, lactate, pyruvate, glutamate, FMN]	Microdialysate glucose and lactate correlate with L-GrAFT, cold ischemia time, macrovesicular steatosis, weight gain during D-HOPE, perfusate FMN

Finally, the active metabolic state of the NMP-perfused liver provides a unique opportunity to push the boundaries of organ reconditioning by delivering therapies directly to the perfused organ, optimizing drug dosages and limiting in vivo toxicity [1]. By this approach, ECD livers that are currently considered untransplantable after viability assessment could be repaired during MP and possibly transplanted. NMP has already been demonstrated as a suitable platform for this purpose and several therapeutic interventions have already been investigated, including defatting strategies, gene delivery and cell-based regeneration [17].

### 3.2. Mesenchymal Stem Cells and MSC-Derived Products in Liver Disease

In the field of regenerative medicine, MSCs are one of the most widely studied cell types, with more than 1000 ongoing trials investigating their potential clinical applications [40]. Given this increasing interest in MSCs as potential therapeutics for several human diseases, and the consequent need for standardization of production protocols, a universal definition for MSCs was proposed with the following criteria [41]: (1) adherence to plastic, (2) specific surface antigen expression (positive for CD105, CD73 and CD9, and negative for CD45, CD34, CD14 or CD11b, CD79a or CD19 and HLA class II) and (3) multipotent differentiation potential into osteoblasts, adipocytes and chondroblasts in vitro.

Although the exact mechanism of their function still remains a matter of research, it has emerged that MSCs play important immunomodulatory and regenerative roles through the activation of paracrine mechanisms [42]. More specifically, MSCs can modulate inflammation and promote tissue repair by releasing cytokines, growth factors and extracellular vesicles (EVs) that enhance the endogenous responses to injury [43]. Tissue repair is also mediated by the ability of MSCs to generate differentiated functioning cells that can directly repopulate the injured site, as in the case of MSC-derived hepatocyte-like cells [44,45,46].

In the setting of liver disease, the beneficial effects of MSCs have been demonstrated in a large number of preclinical studies that investigated their effects in both acute and chronic disease models [46,47,48,49,50,51], eventually leading to their administration to humans suffering from cirrhosis and liver failure [52] (Table 2).

In 2006, Herrera et al. isolated a multipotent cell line from adult human livers, named adult-derived human liver stem cells (HLSCs) [53]. HLSCs expressed typical MSC markers (CD29, CD73, CD44 and CD90) and stem cell markers (vimentin and nestin), but not hematopoietic stem cell markers (CD34, CD45, CD117 and CD133), and were partially committed to the hepatic lineage, as supported by the expression of albumin, alpha-fetoprotein and, in a small percentage of cells, cytokeratin-8 and cytokeratin-18 [53]. Furthermore, HLSCs differentiated to mature hepatocytes when cultured in specific culture conditions [53,54] and exerted immunomodulatory properties [55]. To date, several animal studies of liver injury confirmed the regenerative potential of HLSCs [56,57,58] and the first-in-man application resulted in their safe intrahepatic administration in infants with inherited neonatal-onset hyperammonemia [59].

As mentioned before, there is growing evidence that the impact of MSCs mainly relies on their secretome rather than on their differentiation into target cells [42]. On this basis, many groups focused on the development of cell-free approaches to potentially avoid some stem cell-based therapy limitations, such as availability, poor engraftment potential, immune rejection and risk for malignant transformation [60].

MSC-derived conditioned medium (MSC-CM) contains a high number of cytokines and paracrine factors that produced comparable effects to MSC-based therapies. In fact, MSC-CM was able to attenuate inflammation, apoptosis and necrosis while promoting cell proliferation in different models of acute liver injury [61,62]. Moreover, MSC-CM effectively improved mitochondrial function and insulin resistance and reduced collagen deposition in mice chronic models of non-alcoholic fatty liver disease and cholestatic fibrosis, respectively [63,64]. Most MSC-CM biological effects, however, are mediated by the EVs it contains. 

EVs are a heterogeneous group of membrane-enclosed structures of varying sizes and different origins that are released from almost every cell type [65]. They act as paracrine factors, carrying various functional molecules, mostly proteins and RNAs, that can reveal the state of the parent cells and contribute to inter-cellular communication under both physiological and pathological conditions. These characteristics have prompted the use of EVs as diagnostic markers and therapeutic targets, also, given that, as compared to MSCs, they can be more easily stored and administered, they act more similarly to a drug than a cell-based therapy [66]. EVs can be categorized by size as (1) exosomes (30–150 nm diameter), which are released from the fusion of multivesicular bodies with the plasma membrane, (2) large EVs, such as microvesicles, also called outer membrane vesicles, ectosomes or shedding vesicles (100 to 1000 nm diameter), which are generated by plasma membrane budding, and (3) apoptotic bodies, which fall off during the process of cell apoptosis (50 to 5000 nm diameter) [65,67]. Moreover, EVs can be also classified based on their content, biogenesis, functions or secretory origins, and this wide variety of subcategories clearly demonstrates the need for standardized production and characterization protocols that make their therapeutic use feasible and reproducible [68].

In the setting of liver diseases, animal studies demonstrated that MSC-derived EVs (MSC-EV) can inhibit apoptosis, enhance hepatocyte function, promote angiogenesis and proliferation and reduce the inflammatory response to IRI [69,70,71], making them suitable therapeutic candidates not only for acute and chronic liver diseases [72], but also for the transplantation setting. Similar results have been obtained with HLSC-derived EVs (HLSC-EV), which have been shown to promote liver regeneration in rats undergoing subtotal hepatectomy [73], attenuate fibrosis [74] and reduce hepatic IRI [75]. 

**Table 2:** Schematic overview of experimental studies applying MSCs and MSC-derived products to treat acute and/or chronic liver injury.

Author	Treatment	Model	Administration	Dose	Outcomes
Herrera et al., 2006 [53]	HLSC	Acetaminophen-induced ALF (mouse)	Systemic intravenous	2 × 10^5^ cells	↑ Regeneration
Herrera et al., 2010 [73]	HLSC-EV	70% hepatectomy (rat)	Systemic intravenous	30 µg EVs	↓ ALT, AST, apoptosis ↑ albumin, proliferation
Herrera et al., 2013 [56]	HLSC and HLSC-CM	GalN/LPS-induced ALF (mouse)	Systemic intravenous/intrahepatic/intraperitoneal	2 × 10^6^ cells (IV) 30 × 10^6^ cells (IP) 0.5–0.2 × 10^6^ cells (IH) Concentrated CM (IP)	↑ Survival, regeneration ↓ AST, ALT, ammonium, apoptosis
Stock et al., 2014 [45]	Hepatocyte-like cells derived from human MSC	Acetaminophen-induced ALF (mouse)	Intrasplenic	1 × 10^6^ hepatocyte-like cells	↓ Inflammation, apoptosis ↑ Regeneration
Haga et al., 2017 [69]	Human and mouse BM-MSC-EV	GalN /TNF-α-induced ALF (mouse)	Systemic intravenous/intraperitoneal	2 × 10^10^ EVs	↑ Survival ↓ ALT, AST, ALP (hMSC-EV), direct bilirubin (mMSC), inflammation, apoptosis
Haga et al., 2017 [70]	Mouse BM-MSC-EV	Hepatic IRI (mouse)	Systemic intravenous	2 × 10^10^ EVs	↓ ALT, AST, ALP, necrosis, apoptosis, inflammation
Qu et al., 2017 [47]	Human BM-MSC	Acute HBV infection (mouse)	Systemic intravenous	1 × 10^6^ cells	↓ ALT, IL-1β, IL-6, TNF-α, CCL3 ↓ NK cells activity
Chen et al., 2018 [61]	BM-MSC co-cultured with hepatocytes-CM	GalN/LPS-induced ALF (rat)	N/A	CM 3 times per day for 3 consecutive days	↑ ALF recovery, survival ↓ ALT, AST, bilirubin, necrosis
Hwang et al., 2019 [48]	Lipid-conjugated heparin-coated human ADSC	Acetaminophen-induced ALF (mouse)	Systemic intravenous	4 × 10^5^ cells	↑ Biodistribution, regeneration ↓ AST, ALT, macrophage CYP2E1
Luo et al., 2019 [49]	Mouse BM-MSC	Chronic liver fibrosis (mouse)	Systemic intravenous	5 × 10^5^ cells	↓ AST, ALT, IFN-γ, TNF-α, IL-6, IL-12b, TGF-β1, α-SMA, collagen-1, and collagen-4 ↑ MMP13, IL-10, caspase 3
Famulari et al., 2020 [58]	HLSC	Crigler Najjar Syndrome type I (mouse)	Intrahepatic	1 × 10^5^ cells	↑ Survival ↓ Bilirubin, brain injury
Rostom et al., 2020 [66]	Rat BM-MSC or MSC-EV	CCl_4_-induced chronic liver fibrosis (mouse)	Systemic intravenous	80 µg EVs protein/rat 1 × 10^6^ cells 3 × 10^6^ cells	↓ ALT, AST ↑ Albumin in MSC groups ↓ Collagen deposition in 1 × 10^6^ MSC and EV groups
Cai et al., 2020 [50]	Human umbilical cord-derived MSC labelled with melanin nanoparticles	Acetaminophen-induced ALF (mouse)	Systemic intravenous	5 × 10^5^ cells	Effective long-term in vivo tracking ↓ AST, ALT
Bruno et al., 2020 [74]	HLSC-EV	NASH (mouse)	Systemic intravenous	2.5 × 10^10^ EVs 5 × 10^10^ EVs	↓ ALT, inflammatory and pro-fibrotic gene expression ↑ albumin
Calleri et al., 2021 [75]	HLSC-EV	Hepatic IRI (mouse)	Systemic intravenous	3 × 10^9^ EVs 7.5 × 10^9^ EVs	↓ ALT, LDH, tissue injury, inflammation
Yang et al., 2021 [63]	Human umbilical cord-derived MSC-CM	NAFLD (mouse)	Systemic intravenous	CM every 3 days for 2 months	↑ Mitochondrial function ↓ ALT, AST, steatosis, inflammation, apoptosis
Pinheiro et al., 2021 [64]	Mouse adipose tissue-derived MSC-CM	Cholestatic liver fibrosis (mouse)	Intraperitoneal	CM at 5th and 6th day after bile duct ligation	↓ ALT, AST, ALP, collagen deposition, inflammation
Ma et al., 2022 [51]	Human umbilical cord-derived MSC and mouse adipose-derived MSC	CCl_4_-induced ALF and chronic liver fibrosis (mouse)	Systemic intravenous	5 × 10^5^ cells	Acute injury: ↓ AST, ALT, TNF-α, IL-1β, MCP-1, NK activity Chronic injury: limited effects and ↑ NK activity
Abo-Aziza et al., 2022 [46]	Rat BM-MSC and hepatogenic cells derived from BM-MSC	Acute aflatoxicosis (rat)	Systemic intravenous/intrahepatic	2 × 10^6^ cells at 2-week intervals	↓ TNF-α, IL-4, AST, ALT ↑ Antioxidant enzymes

### 3.3. Current Application of MSCs and Derived Products during Machine Perfusion of the Liver

The attractive immunomodulatory and regenerative effects of MSCs and the possibility to maintain the liver metabolically “alive” outside the human body using NMP stimulated the idea of delivering stem cell therapies directly to the perfused graft, exploiting the practical and biosafety advantages offered by this unique platform (Table 3). The first experiments that successfully proved this concept were performed on lungs and kidneys, leading to further pre-clinical studies that confirmed the ability of MSCs and MSC-derived products to recover renal and pulmonary function during MP [17]. 

Sasajima et al. [76] were the first to successfully recondition rat livers procured after circulatory death (DCD) with MSCs administered during NMP. In their experiment, the administration of swine adipose tissue-derived stem cells at the start of perfusion with blood-free perfusate was associated with increased bile production and preserved sinusoidal and hepatocellular morphology after 2 h of NMP. 

Based on these preliminary results, several experiments were performed by the Tianjin group using bone marrow-derived MSCs (BM-MSCs) to repair rat livers exposed to 30 min of warm ischemia before NMP [77,78,79,80,81,82,83,84]. In all these studies, the treatment was associated with less transaminases released into perfusate, increased bile production and improved histology [77,78,79]. Interestingly, authors provided a consistent mechanistic explanation to these protective effects, showing that BM-MSCs can regulate different pathways of the IRI cascade. Oxidative stress, mitochondrial injury and macrophage activation, all key elements of the ischemic and early post-reperfusion response, were reduced in the treated groups, as well as endothelial activation and sinusoidal microcirculation impairment, which occur during the late phase of IRI [77,78]. Furthermore, BM-MSCs downregulated ROS and free Fe^2+^ release, suggesting a protective role against ferroptosis, a type of programmed cell death mediated by iron-dependent accumulation of lipid peroxides [79]. The promising potential of this treatment was confirmed in a rat model of DCD liver transplantation, where the combination of BM-MSCs and NMP increased post-transplant survival rate by 10% compared to NMP alone, and reduced biliary injury, as confirmed by higher CK19 expression and better bile ducts [80]. The same group demonstrated that, if transfected with the heme oxygenase-1 (HO-1) gene, BM-MSCs significantly increase their protective effects. HO-1 is an enzyme involved in heme metabolism that can reduce iron overload and ROS production, resulting in improved cell viability [85]. As a result, compared to BM-MSCs, HO-1-modified BM-MSCs (HO-1/BM-MSCs) administered during NMP were more effective in reducing tissue injury and animal mortality [81,82,83,84]. At the molecular level, HO-1/BM-MSCs downregulated HMGB1 and its downstream TLR4/NF-κB pathway, resulting in a significant reduction of pro-inflammatory cytokines levels [81]. Moreover, focusing on the biliary system, HO-1/BM-MSCs activated peribiliary glands cells via the Wnt pathway, effectively repairing bile ducts after DCD liver transplantation [84]. In a murine model of acute rejection, HO-1/BM-MSC treatment was associated with increased liver viability and animal survival, being as effective as calcineurin inhibitors in reducing cellular rejection features. Among the possible mechanism of protection, authors observed that the treated livers had less INF-γ expression and NK and CD8+ T cells infiltration [82], together with reduced dendritic cells and CD4+ T cells activation [83]. These data are of extreme interest, as they confirm the immunomodulatory activity of MSCs and, along with the advantages of their ex vivo administration, make them potential candidates to favor progressive immunosuppression withdrawal and development of operational tolerance after LT [86]. 

Verstegen et al. successfully translated this technique into a large animal model [87]. They delivered human MSCs to pig livers using HMP, showing a wide range and distribution of MSCs throughout the organ after 30 min of perfusion. During 4 subsequent hours of NMP, perfusate analysis confirmed the ability of MSCs to modulate key inflammatory genes in a paracrine way. A landmark study by Laing et al. eventually proved the feasibility of this approach in humans [88]. In this study, multipotent adult progenitor cells (MAPCs) were infused directly into the right liver lobe, either via the hepatic artery or the portal vein, during NMP of six discarded human livers. Confocal microscopy demonstrated MAPC engraftment, with artery-infused cells being able to transmigrate across the vascular endothelium. Multiplex assay and proteomics analyses on NMP perfusate revealed the immunomodulatory effect associated with MAPC infusion. 

Compared to stem cell-based treatments, EVs could be advantageous in terms of genetic stability, storage conditions and administration route, also during dynamic preservation, where the potential detrimental effects that NMP components may have on cellular membranes could be avoided using cell-free approaches [89,90]. In a model of hypoxic liver NMP, our group has demonstrated the feasibility of delivering EVs to hepatocytes during NMP [91,92]. HLSC-EV were administrated at perfusion start and were detected in the hepatocytes after 4 h of NMP, as confirmed by epifluorescence microscopy. Cytolysis, tissue injury markers, as well as HIF-1α and TGF-β1 overexpression were significantly reduced in the treated group, suggesting a protective effect against hypoxia. To further investigate the role of HLSC-EV in a more clinically relevant scenario, we set up an NMP model of DCD rat liver characterized by prolonged warm ischemia time, mimicking the high-risk scenario of clinical DCD LT in Italy [93]. HLSC-EV, which were administrated during NMP at two different doses, were effectively uptaken by the liver and significantly improved cytolysis, pH self-regulation and phosphate utilization. Only the higher dose was associated with higher bile production, lower resistance, reduced necrosis and enhanced proliferation, suggesting a dose-effect relationship.

Since a metabolically active organ is generally considered a prerequisite to apply cell-based therapies, NMP has more frequently been used for such purposes. However, some groups have achieved successfully delivering EVs at low temperatures during kidney HMP, demonstrating their uptake and suggesting a beneficial biological effect [94,95]. Still, the mechanisms of engraftment and biological activity in hypothermic conditions have been scarcely studied, and further investigations supporting this approach are required.

**Table 3:** Schematic overview of preclinical studies investigating MSC-based therapies administered during dynamic liver preservation.

Author	Model	Treatment	Dose	Duration	Perfusate	Mechanism	Outcomes
Sasajima et al., 2018 [76]	30 min-DCD Rat	Swine adipose MSC	2 × 10^5^ 1 × 10^6^	2 h NMP	Krebs–Henseleit bicarbonate buffer (~30–40 mL)	NA	**↑** Bile production ↓ Sinusoidal space narrowing and hepatocellular vacuolization
Rigo et al., 2018 [91]	Rat	Human HLSC-EV	5 × 10^8/^g liver	4 h NMP	Williams E medium, glucose, penicillin, streptomycin, glutamine, insulin, heparin, bicarbonate (50 mL) Full rat blood (20 mL)	↓ HIF-1α, TGF-β1	↓ AST, LDH ↓ Suzuki’s scores, apoptotic cells
Yang et al., 2020 [77]	30 min-DCD Rat	Rat BM-MSC	1 × 10^7^	8 h NMP	DMEM/F12, FBS, penicillin, streptomycin, heparin, insulin, dexamethasone (60 mL) Full rat blood (20 mL)	↓ Macrophage activation ↓ Endothelial activation (↓ICAM-1, VCAM-1, vWF) **↑** Microcirculation perfusion (↓ ET-1, **↑** eNOS)	↓ AST, ALT, ALP, lactate **↑** Bile production ↓ Suzuki’s scores, apoptotic cells ↓ Mitochondrial damage
Yang et al., 2020 [78]	30 min-DCD Rat	Rat BM-MSC	1–3 × 10^7^	8 h NMP	DMEM/F12, FBS, penicillin, streptomycin, heparin, insulin, dexamethasone (60 mL) Full rat blood (20 mL)	↓ Oxidative stress (↓MPO, MDA, **↑** GSH) ↓ Expression of JNK-NF-kB signaling **↑** Expression of AMPK signaling	↓ AST, ALT, lactate **↑** Bile production ↓ Suzuki’s scores, apoptotic cells ↓ Mitochondrial damage
Cao et al., 2020 [81]	30 min-DCD Rat	Rat HO-1 BM-MSC Rat BM-MSC	1.5–3 × 10^7^	4 h NMP	DMEM/F12, FBS, penicillin, streptomycin, heparin, insulin, dexamethasone (60 mL) Full rat blood (20 mL)	↓ IL-1β, IL-6, TNFα, HMGB1 ↓ Expression of JNK-NF-kB signaling	↑ Post-transplant survival ↓ Biliary injury (↑ CK19+ cells) ↑ Post-transplant survival ↓ AST, ALT, ALP, gGT ↓ Biliary injury (↑ CK19+ cells) ↓ Suzuki’s scores
Verstegen et al., 2020 [87]	15–45 min DCD Pig	Human BM-MSC	5–10 × 10^6/^kg liver	30 min HOPE 4 h NMP	HOPE: University of Wisconsin solution NMP: autologous pig full blood	↑ IL-6, IL-8	Effective hepatic uptake Immunomodulation
Laing et al., 2020 [88]	Discarded human livers	Human MAPC	50 × 10^6^	6 h NMP	Packed human red blood cells, human albumin, heparin, sodium bicarbonate, calcium gluconate, vancomycin, gentamicin, epoprostenol, aminoplasmal, dextrose	**↑** IL-4, IL-5, IL-6, IL-8, 10, MCP-1, SDF-1α, IL-1β, GM- CSF	Effective hepatic uptake Immunomodulatory effects
Sun et al., 2021 [79]	30 min-DCD Rat	Rat BM-MSC	1–3 × 10^7^	6 h NMP	DMEM/F12, FBS, penicillin, streptomycin, heparin, insulin, dexamethasone (60 mL) Full rat blood (20 mL)	↓ Oxidative stress and ferroptosis (↓ MDA and PTGS2, **↑** GSH and GPX4) ↓ Autophagy (**↑** FTH1 and P62)	↓ AST, ALT **↑** Bile production ↓ Suzuki’s scores
Cao et al., 2021 [82]	30 min-DCD Rat	Rat HO-1 BM-MSC Rat BM-MSC	1 × 10^7^	Not reported	DMEM/F12, FBS, penicillin, streptomycin, heparin (60 mL) Full rat blood (20 mL)	↓ NK-T and T-CD8+ infiltration ↓ IFN-γ, TNF-α, IL-2	**↑** Post-transplant survival ↓ Scute cellular rejection ↓ AST, ALT, ALP, GGT, bilirubin ↓ Apoptotic cells
Tian et al., 2021 [84]	30 min-DCD Rat	Rat HO-1 BM-MSC Rat BM-MSC	1–3 × 10^7^	4 h NMP	DMEM/F12, FBS, penicillin, streptomycin, heparin, insulin, dexamethasone (60 mL) Full rat blood (20 mL)	**↑** Expression of Wnt signaling	**↑** Post-transplant survival ↓ AST ALT, ALP, GGT, bilirubin ↓ Bile ducts integrity ↓ Apoptosis and **↑** proliferation of peribiliary glands cells
De Stefano et al., 2021 [93]	60 min-DCD Rat	Human HLSC-EV	5 × 10^8/^g liver 25 × 10^8/^g liver	6 h NMP	Williams E medium, penicillin, streptomycin, glutamine, insulin, heparin, bicarbonate, taurocholic acid (100 mL) Packed human red blood cells (50 mL)	NA	↓ AST, ALT, phosphates, ↓ Total HCO_3_^−^ need **↑** Bile production (high dose only) ↓ Necrosis and **↑** proliferation (igh dose only) ↓ Vascular resistance (high dose only)
Wu et al., 2022 [83]	30 min-DCD Rat	Rat HO-1 BM-MSC Rat BM-MSC	1 × 10^7^	4 h NMP	DMEM/F12, FBS, penicillin, streptomycin, heparin, insulin, dexamethasone (60 mL) Full rat blood (20 mL)	↓ Dendritic cells maturation ↓ T-CD4+ infiltration ↓ IFN-γ, TNF-α, CCL-2, CXCL-9, CXCL-10	↑ Post-transplant survival ↓ Acute cellular rejection ↓ AST, ALT, ALP, bilirubin ↓ Apoptotic cells

## 4. Future Perspectives and Conclusions

The rise of MP technology as a preservation modality alternative to SCS introduced a new era in LT, offering the unique possibility to increase ECD utilization and improve transplant outcomes by supporting graft metabolism ex vivo. The current scenario includes a plurality of MP approaches that differ in timing, technical modalities and logistical implications that altogether provide transplant surgeons with the opportunity to objectively assess organ quality and prolong preservation times [1,25]. 

MSCs can regulate IRI and promote tissue repair and regeneration. Solid preclinical evidence supports their application in liver diseases, and many ongoing clinical trials will likely validate their use as a standard-of-care in the future. Products derived from MSCs (such as MSC-CM and MSC-EV) preserve the therapeutic potential of their originating cells, but avoid at the same time most of the safety concerns associated with live cell treatments [52]. 

Although not strictly related to MSCs, organoids deserve a brief mention as their use in regenerative medicine has enormous potential. Organoids are 3D multicellular spherical structures made of one or more cell types that can mimic in vivo conditions by reproducing the dynamic regulation of signaling pathways and paracrine signals that underlie cell-to-cell and cell-to-matrix interactions [96]. Based on these characteristics, organoid technology was applied in disease modelling and drug screening, but recently there has been a growing interest in organoids transplantation as a way to repair injured tissues. Indeed, several animal models of hepatobiliary injury demonstrated the effective engraftment of organoids generated from stem cells [97] or, more promisingly, from differentiated cells such as primary hepatocytes and cholangiocytes. Sampaziotis et al. [98] performed a groundbreaking study using NMP as a platform to inject cholangiocyte organoids into intrahepatic bile ducts of discarded human livers. Supported by preliminary murine experiments [99], authors demonstrated that organoids derived from extrahepatic cholangiocytes had the plasticity to adapt to the intrahepatic environment and effectively repair damaged bile ducts.

The parallel development of MP and cell-based therapies has paved the way for the combination of these technologies. Besides reducing IRI and improving the results of LT, MP creates a window of opportunity during which organ treatments can be administered. Administration of cell-based therapies during ex situ perfusion has several advantages, including the possibility of optimizing dosage, while avoiding the downsides of systemic administration. The possibility of prolonging NMP for several days, pioneered by the Zurich group [100,101,102], may be key in extending the therapeutic window for organ regeneration therapies to be effective [103,104,105]. Further pre-clinical studies investigating adequate dosing, biosafety and short-term outcomes are still needed to address the translational gap. 

## Data Availability

Not applicable.
